# The Mysteries of Capsaicin-Sensitive Afferents

**DOI:** 10.3389/fphys.2020.554195

**Published:** 2020-12-16

**Authors:** Michael J. M. Fischer, Cosmin I. Ciotu, Arpad Szallasi

**Affiliations:** ^1^Center of Physiology and Pharmacology, Medical University of Vienna, Vienna, Austria; ^2^1st Department of Pathology and Experimental Cancer Research, Semmelweis University, Budapest, Hungary

**Keywords:** capsaicin, TRPV1 receptor, ion channel, pain, sensory neuron, inflammation, thermoregulation

## Abstract

A fundamental subdivision of nociceptive sensory neurons is named after their unique sensitivity to capsaicin, the pungent ingredient in hot chili peppers: these are the capsaicin-sensitive afferents. The initial excitation by capsaicin of these neurons manifested as burning pain sensation is followed by a lasting refractory state, traditionally referred to as “capsaicin desensitization,” during which the previously excited neurons are unresponsive not only to capsaicin but a variety of unrelated stimuli including noxious heat. The long sought-after capsaicin receptor, now known as TRPV1 (transient receptor potential cation channel, subfamily V member 1), was cloned more than two decades ago. The substantial reduction of the inflammatory phenotype of *Trpv1* knockout mice has spurred extensive efforts in the pharmaceutical industry to develop small molecule TRPV1 antagonists. However, adverse effects, most importantly hyperthermia and burn injuries, have so far prevented any compounds from progressing beyond Phase 2. There is increasing evidence that these limitations can be at least partially overcome by approaches outside of the mainstream pharmaceutical development, providing novel therapeutic options through TRPV1. Although ablation of the whole TRPV1-expressing nerve population by high dose capsaicin, or more selectively by intersectional genetics, has allowed researchers to investigate the functions of capsaicin-sensitive afferents in health and disease, several “mysteries” remain unsolved to date, including the molecular underpinnings of “capsaicin desensitization,” and the exact role these nerves play in thermoregulation and heat sensation. This review tries to shed some light on these capsaicin mechanisms.

## Capsaicin as a Tool Before TRPV1 Was Discovered

Natural products like capsaicin afford a unique tool to dissect important physiological pathways. The recognition that consuming the fruits of capsicum plants evokes a characteristic “hot” burning sensation in the human tongue and oral mucosa is probably as old as the domestication and cultivation of these plants (going back to 8,000 years in South America) (Perry et al., 2007). And here is the first great “mystery” of capsaicin-sensitive afferents: how come that the very same pungent sensation that repels herbivores (like deer) from eating the capsicum pods is found pleasurable by so many humans? Recently, experts tried to solve this puzzle in the journal Temperature with such fascinating explanations like the cooling effect of spicy food (capsaicin as “natural air-conditioner”), the food-preserving, anti-microbial action of capsicum (capsaicin as “refrigerator”), or simply the “masochism” of chili-lovers (Szallasi, 2016). Nonetheless, the human fondness of, or aversion to hot pepper is probably far more complex than these models imply (Byrnes and Hayes, 2013).

From *Capsicum*, capsaicin was first isolated in 1846 (Thresh, 1846), and its chemical structure determined in 1919 (Nelson, 1919). In 1912, Wilbur Scoville invented his human tongue-based scale to measure the “hotness” of pepper extracts (Gmyrek, 2013; Sweat et al., 2016). Ever since, capsaicin has remained a reference agonist in sensory pharmacology (Szolcsányi, 1984; O’Neill et al., 2012). The early capsaicin findings were detailed elsewhere (Szolcsányi, 1984, 2004) and only the most important milestones are listed here.

The effect of capsaicin on thermoregulation was first noted 150 years ago when hot pepper extract applied to the stomach of dogs produced a fall in rectal temperature (Högyes, 1878). With regard to pain and inflammation, Nicholas (Miklós) Jancsó made the astute observation that capsaicin evoked strong and persistent “desensitization” after exposure to the rat cornea, skin, and airways (Jancsó and Jancsó, 1949; Jancsó et al., 1967).

Capsaicin research gathered speed in the 1970s. Electrical nerve stimulation was shown to cause neurogenic inflammation, and this could be ablated by prior high dose capsaicin exposure. Similarly, responsiveness to mustard oil or cigarette smoke (both turned out to activate TRPA1) or the sodium channel modulator veratridine was reduced. Since these substances only partially cross-desensitize, this indicated a prolonged silencing of the capsaicin-sensitive neurons, and an overlap with the respective receptor populations (Szolcsányi et al., 1975). In contrast, mechanical sensitivity was largely untouched by high concentrations of capsaicin. In the 1980s, researchers acquired important new tools to study capsaicin-sensitive afferents, including ruthenium red as the first (though non-selective) capsaicin antagonist (Maggi et al., 1988), capsazepine as the first synthetic and somewhat selective capsaicin antagonist (Urban and Dray, 1991), and resiniferatoxin, an ultrapotent capsaicin analog with a unique spectrum of actions (Szallasi and Blumberg, 1989).

Much of our early knowledge of capsaicin-sensitive pathways came from the desensitization experiments. It cannot be exphasized enough that the literature uses the term “capsaicin desensitization” loosely, in an ill-defined manner. By “desensitization”, some investigators mean a fully reversible capsaicin-induced refractory state, whereas others use it more broadly to include irreversible changes due to neuronal death (Szallasi and Blumberg, 1999).

It is not clear whether the reversible and irreversible refractoriness following capsaicin (or resiniferatoxin) administration reflect quantitative or qualitative differences. For example, in the human neurogenic bladder, topical resiniferatoxin induces a long lasting (up to several months), but fully reversible, increase in the cystoscopic volume at which the voiding urge is activated (Cruz et al., 1997), without causing any noticeable changes in the bladder biopsies at the light or electron microscopic level (Silva et al., 2001). By contrast, resiniferatoxin applied to the bodies of capsaicin-sensitive neurons causes irreversible changes by selectively ablating these cells *via* Ca^2+^ influx-mediated cytotoxicity (Karai L. et al., 2004).

Using resiniferatoxin as a “molecular scalpel” to achieve permanent analgesia has a clear therapeutic potential (Iadarola and Gonnella, 2013). Indeed, intrathecal resiniferatoxin is already undergoing clinical trials in severe osteoarthritic pain (www.clinicaltrials.gov, NCT 04044742 Sorrento Therapeutics Inc., 2020), and in cancer patients with chronic intractable pain (www.clinicaltrials.gov, NCT 00804154, 2020). Furthermore, a site-specific (intraarticular) trans-capsaicin (CNTX-4975) injection has shown promise in osteoarthritis knee pain (Stevens et al., 2019); the results of two on-going phase 3 trials are expected soon.

Calcium is clearly a key player in capsaicin actions (Wood et al., 1988; Bevan and Szolcsányi, 1990). Calcium overload also underlies both desensitization and neurotoxicity by resiniferatoxin (Olah et al., 2001). Of note, capsaicin and resiniferatoxin differ in the kinetics of the Ca^2+^ influx that they evoke: the current is fast and rapidly normalizing for capsaicin, whereas it is sustained and long-lasting for resiniferatoxin (Szallasi et al., 1999). This difference is so striking that it even led to the proposal (later discredited by the cloning of the vanilloid receptor, TRPV1) of distinct vanilloid receptors mediating capsaicin (C-type) and resiniferatoxin (R-type) actions, respectively (Bíró et al., 1998). One may argue that there is a fine and ill-defined line that separates reversible desensitization from irreversible toxicity. This may involve the route of application (peripheral nerve terminal *versus* cell body), the kinetics of Ca^2+^ influx, the phosphorylation state of the receptor protein, as well as other, as yet unidentified, mechanisms.

In rat models of chronic neuropathic pain, there appears to be a genetic reprogramming in injured nociceptive neurons in which pain-promoting mechanisms are up-regulated: it was referred to as messenger plasticity (Hökfelt et al., 1994). Interestingly, resiniferatoxin administration was found to change the phenotype of sensory neurons the opposite way, by down-regulating the expression of substances know to promote pain (e.g., substance P), and by up-regulating endogenous pain-countering compounds, like galanin (Szallasi, 1996). Importantly, resiniferatoxin also blocked the neuronal synthesis of its own receptor. These resiniferatoxin-induced neurochemical changes, collectively referred to as “vanilloid-induced messenger plasticity,” were fully reversible, and their recovery coincided with the return of pain sensitivity (Szallasi and Blumberg, 1999).

Early experiments already showed that acute systemic exposure to capsaicin reduced body temperature (hypothermia), and, after ablation of the sensitive neurons, it rendered animals unable of behavior saving themselves from overheating (hyperthermia) (Jancsó-Gábor et al., 1970). These observations could have served as an early warning of the thermoregulatory side-effects of TRPV1 antagonists that, somehow, had to be rediscovered later. It should be mentioned here that the undesirable effects on the body temperature have been minimized in the second generation TRPV1 antagonists (Gomtsyan et al., 2015), but whether this also reduced potential therapeutic uses in parallel is not yet known. Site and mechanism of the body temperature regulation fall beyond the scope of this review and are discussed elsewhere (Garami et al., 2018).

In the 1970s, based on the fairly strict structure-activity-requirements for capsaicin-like activity, Szolcsányi and coworkers postulated the existence of a specific capsaicin receptor (Szolcsányi and Jancsó-Gábor, 1975). In 1990, specific binding of resiniferatoxin provided the first biochemical proof for the existence of this receptor, called the vanilloid receptor VR1 (Szallasi and Blumberg, 1990), and [^3^H]resiniferatoxin autoradiography was used to visualize the expression of this receptor in several species, including man (Szallasi, 1995). In patch-clamped *Xenopus oocytes*, a capsaicin-induced current was observed following the injection of RNA extracted from rat sensory neurons (Szallasi, unpublished observations). With this, the hunt for the capsaicin receptor was on.

## The Discovery of TRPV1

In 1997, the laboratory of David Julius was the first to identify the rat capsaicin (vanilloid) receptor *via* an expression cloning strategy that took advantage of the Ca^2+^ conductance (Caterina et al., 1997). The human isoform showed largely similar properties (Hayes et al., 2000). The availability of a plasmid led to rapid characterisation of the properties of the receptor protein, including pharmacological and biophysical properties. Importantly, the capsaicin receptor turned out to be a transient receptor potential (TRP) channel. Within the TRP superfamily, the capsaicin receptor as TRPV1 is the founding member of the now populous TRPV (vanilloid) subdivision, TRPV1 to TRPV6 (Clapham, 2003).

Regarding the “transient nature” of TRP channels, it is really a misnomer explained by the history of this naming convention. In 1969, a drosophila eye mutant labeled trp responded to lasting light stimulation with a transient depolarizing after-potential instead of the normal prolonged response (Cosens and Manning, 1969). The respective wild-type *trp* gene was isolated and could rescue this phenotype (Montell et al., 1985). So, the wild-type channel in fact causes a persistent (and not transient) current; nevertheless, this ion channel family now bears the name “transient”. This is in contrast to many ion channels, which fully adapt when exposed to constant stimulation, and is important to continuously code pain for a persistent stimulus.

Subsequently, the Julius laboratory generated and characterized the *Trpv1* knockout mouse, which misses exon 13 that codes mainly for the pore loop and transmembrane domain 6. These animals looked normal, but lacked responses to capsaicin, showed normal responses to noxious mechanical stimuli, and expressed minimal inflammatory thermal hyperalgesia (Caterina et al., 2000). This phenotype was consistent with that of the capsaicin-desensitized rodents and rendered TRPV1 immediately as an attractive pharmacological target. In addition, which could have been an early warning of the burn injury in patients on TRPV1 antagonists, there was a reduced pain-related behavior to noxious heat, in particular at higher temperatures (Caterina et al., 2000). A separately generated *Trpv1* knockout mouse led to similar results (Davis et al., 2000).

Moreover, the crystal structure of the TRPV1 protein was largely solved by cryo-electron microscopy (Liao et al., 2013), including an open conformation (Cao et al., 2013) and the transmembrane part in a chemically more native lipid nanodisc environment (Gao et al., 2016).

## TRPV1 as a Sensor

TRPV1 is multimodally-gated channel, activated in concert by both physical and chemical stimuli. Importantly, from a “native state”, the channel’s sensitivity can be substantially increased by chemical modification, e.g., phosphorylation (Numazaki et al., 2002; Szallasi et al., 2007; Utreras et al., 2013; Nagy et al., 2014). Consequently, the adjustable working range of the responsiveness is surprisingly broad. Furthermore, different modes of activation can act in an additive or supraadditive fashion, and thus subthreshold stimuli acting together may reach the activation threshold.

TRPV1 is activated by heat (Caterina et al., 1997) with a threshold of just above 40 °C (Zhang et al., 2018): this is not far from the human heat pain threshold of about 40 °C (Yarnitsky et al., 1995; Rolke et al., 2006). The ion channel pore domain is responsible for the sensitivity to heat (Zhang et al., 2018). TRPV1 is also activated by acidic pH (Tominaga et al., 1998) with good concentration-dependent coding below pH 6. The biophysics of this activation along with the amino acid residues involved were detailed elsewhere (Jordt et al., 2000; Ryu et al., 2007; Aneiros et al., 2011). The proton-activation of TRPV1 is complicated by the proton-induced inhibition, which applies to most currents, including proton-activated ones (Fischer et al., 2003; Lee and Zheng, 2015).

Natural pharmacological agonists of TRPV1 include both pungent plant products (e.g., capsaicin, piperine and resiniferatoxin) and painful animal venoms and toxins (from spiders, scorpions, centipedes, snakes, and jelly fish, etc.) (Geron et al., 2017; Chu et al., 2020). In addition to capsaicin, chili peppers contain other less pungent compounds like capsiate, summarized under the term “capsinoids”. Of note, pungent compounds occur in various plant-derived spices like red and black pepper, mustard, horse radish, and wasabi. Although these spices taste similarly, their active ingredients act on different molecular targets, primarily TRPV1 and TRPA1.

The existence of endogenous TRPV1 activators (so-called “endovanilloids”) with physiological or pathophysiological relevance remains putative. Although several endogenous lipids, e.g., anandamide (Zygmunt et al., 1999) and other acylethanolamines (Brito et al., 2014), were reported to activate TRPV1 *in vitro*, this activation was observed at such high concentrations that are unlikely to occur *in vivo*. This is also true for lipid oxidation products (Hwang et al., 2000).

Voltage-dependent gating of TRP ion channels was first shown for TRPM4 (Launay et al., 2002). Voltage also seems to be a cooperative factor in the gating of TRPV1 (Voets et al., 2004). Osmotic activation of TRPV1 was also reported, but this has not been reproduced by others (Nishihara et al., 2011). TRPV1 is regulated by phospholipids, but the details are a controversial issue which was critically reviewed elsewhere (Rohacs, 2015).

Inflammatory sensitisation of TRPV1 is an important mechanism of on-going pain (Fischer et al., 2010; Malek et al., 2015). A fast and large degree of sensitisation can be achieved by phosphorylation of the TRPV1 protein, more so *via* protein kinase C than protein kinase A (Premkumar and Ahern, 2000; Vellani et al., 2001; Bhave et al., 2002; Mandadi et al., 2006; Fischer and Reeh, 2007; Wang et al., 2015). Furthermore, substances generated under inflammatory conditions (e.g., nerve growth factor) may either regulate TRPV1 expression (Amaya et al., 2004; Chu et al., 2011) and or act more directly (Zhang et al., 2005).

The substantial variation in the exact thermal activation threshold of TRPV1 is due to its phosphorylation state (Sugiura et al., 2002; Huang et al., 2006; Li et al., 2014). Conformational changes (sensitisation) by phosphorylation bring the channel closer to its activation state, thereby lowering the activation threshold for agonists. This concept applies not only to chemical agonists but to all modes of activation, including temperature and voltage. Protons and/or capsaicin can act with temperature in a supraadditive fashion (Jordt et al., 2000; Kichko and Reeh, 2004), and this also applies to voltage (Voets et al., 2004). The extent of channel activation by a specific agonist-binding site depends on the agonist, with a partial agonist showing less shift and acting antagonistic to a full agonist. Indeed, this expected phenomenon was demonstrated for iodo-resiniferatoxin (Shimizu et al., 2005). Less expected was the different number of agonist-bound sites for activation: using concatemers with inactivated capsaicin or proton binding sites, it was shown that one capsaicin is sufficient to activate TRPV1, but the same response needs four protons, indicating an agonist-site dependent shift toward activation (Hazan et al., 2015).

Since increased TRPV1 sensitivity might primarily occur as part of local pathology (e.g., local inflammation) (Malek et al., 2015), in contrast to addressing TRPV1 directly (Moran and Szallasi, 2018), selectively targeting sensitisation without affecting the native channel has been investigated as a novel therapeutic approach (Btesh et al., 2013; Fischer et al., 2013; Hanack et al., 2015; Sondermann et al., 2019). It is hoped that side effects that plagued the use of *per os* TRPV1 antagonists can be avoided by this approach.

Continuous or frequently repeated TRPV1 stimulation leads to receptor tachyphylaxis that should be clearly distinguished from capsaicin-induced defunctionalisation of the whole sensory neuron. Tachyphylaxis depends on Ca^2+^ influx, dephosphorylation (Docherty et al., 1996; Mohapatra and Nau, 2005), and association with protein complexes (Por et al., 2013); combined, these effects lead to decreased TRPV1 presence in the plasma membrane. When the Ca^2+^ influx is terminated, the channel is recycled to the plasma membrane from the intracellular depots by shuttle molecules like synaptotagmin 1 (Tian et al., 2019). Of note, a fraction of internalized TRPV1 gets degraded (Sanz-Salvador et al., 2012). Similar to cooperative activation, desensitization by different modalities is also at least partially convergent; for example, desensitization by heat also renders TRPV1 less sensitive to capsaicin (Sánchez-Moreno et al., 2018).

TRPV1 expression is found through the animal kingdom. Species-related differences in TRPV1 function allow a molecular dissection of ion channel biophysics. The Bactrian camel (*Camelus ferus*) and the thirteen-lined ground squirrel (*Ictidomys tridecemlineatus*) both show reduced sensitivity to heat, resisting activation of TRPV1 until 46°C (Laursen et al., 2016). Similarly, chicken (*Gallus gallus*) TRPV1 has an increased activation threshold of around 46°C, but is additionally insensitive to capsaicin (Jordt and Julius, 2002). At the other end of the thermal scale, there are species that have developed a high sensitivity to thermal stimuli such as the axolotl (*Ambystoma mexicanum*) or zebrafish (*Danio rerio*) that have TRPV1 activation thresholds of ∼31°C (Hori and Saitoh, 2020) and ∼33 °C (Gau et al., 2013), respectively. The platypus (*Ornithorhynchus anatinus*) exhibits a lack of heat induced desensitization of TRPV1 in the context of normal heat activation thresholds (Luo et al., 2019), rendering it more susceptible than more evolved species to heat induced damage. Here, the platypus TRPV1 helped to dissect the heat-induced desensitization in mouse TRPV1, where an interaction between the C- and N-termini leads to the rearrangement of the outer pore domain (Luo et al., 2019). In contrast to other modes of activation like heat, capsaicin sensitivity is more evolutionary conserved, notable exceptions being the avian TRPV1 (Jordt and Julius, 2002) and the tree shrew (*Tupaia belangeri chinensis*), the latter with an EC_50_ of 1.9 mM (Han et al., 2018). Medicinal leech (*Hirudo medicinalis*), clawed frog (*Xenopus tropicalis*), and rabbit (*Oryctolagus cuniculus*) TRPV1 all exhibit reduced capsaicin sensitivity, with EC_50_ values of 100, 85, and 15 μM, respectively (Gavva et al., 2004; Ohkita et al., 2012; Summers et al., 2014). Taken together, these observations imply that the acquisition of capsaicin sensitivity was an early event in evolution, and that birds lost their capsaicin sensitivity.

TRPV1 exerts its primary function in the plasma membrane as noxious signal integrator, although a large fraction is always in intracellular compartments with rapid cycling. Although the presence of TRPV1 in the endoplasmic reticulum is well-established (Turner et al., 2003; Gallego-Sandín et al., 2009), the functional relevance of this observation is unclear since the sensitivity of TRPV1 to capsaicin in the endoplasmic reticulum is about ∼100-fold lower than in the plasma membrane. Moreover, Ca^2+^ depletion of the endoplasmic reticulum poses substantial cellular stress which can lead to cell death, and this can be induced by higher capsaicin concentrations than required for TRPV1 activation at the plasma membrane (Karai L. J. et al., 2004).

In the mouse, two splice variants of TRPV1 (missing some exons) have been reported. These variants are not sensitive to capsaicin, but they interact with the full-length channel and act inhibitory in a concentration-dependent manner (Vos et al., 2006; Eilers et al., 2007). The physiological function of these negative variants remains unknown. Alternative splicing of TRPV1 in the trigeminal ganglia allows vampire bats (*Desmodus rotundus)* to detect infrared radiation; the respective short TRPV1 isoform has an activation threshold of about 30 °C (Gracheva et al., 2011), similar to how pit vipers and pythons use TRPA1 in their infrared sensing organs (Gracheva et al., 2010). Similar splicing occurs in cattle (*Bos taurus*), coast moles (*Scapanus orarius*), dogs (*Canis lupus*), and all members of the *Laurasiatheria clade* (a large group of placental mammals), highlighting a mechanism for physiological tuning of thermosensory nerve fibers (Gracheva et al., 2011).

The role of TRPV1 in body weight regulation also remains a mystery. One study reported that *Trpv1* knockout mice remain lean on high-fat diet (Motter and Ahern, 2008), another study found no difference between the body weight of wild-type and *Trpv1* knockout animals (Marshall et al., 2013), and a third study described a mouse which is lean and hyperactive when young, and lazy and fat when becomes old (Wanner et al., 2011).

## The TRPV1-Positive Neuron Population

### TRPV1 Expression

TRPV1 is expressed in a subpopulation of sensory afferents, primarily of the small to medium diameter (Szallasi, 2016). These are mainly slow-conducting, unmyelinated C-fibers, and a subpopulation of thin myelinated A-fibers (Mitchell et al., 2010, 2014; Blivis et al., 2017). The cell bodies of the neurons that give rise to these afferents are located in sensory (e.g., dorsal root and trigeminal) ganglia. These neurons transmit sensory information from the periphery to the dorsal horn of the spinal cord.

The fraction of TRPV1-expressing neurons in sensory ganglia depends on the species, as well as on the location. Expression data are mainly based on protein, mRNA, or reporters. In a study with direct comparison, TRPV1 was expressed in 37% of mouse and 47% of rat dorsal root ganglion (DRG) neurons (Orozco et al., 2001). There are systematic differences between laboratory mouse strains (Ono et al., 2015). A similar TRPV1 expression was found in trigeminal and visceral afferents (Helliwell et al., 1998). Interestingly, TRPV1 mRNA was found in 47% of rat DRG neurons with TRPA1 expression in a subpopulation of these and a mutually exclusive TRPM8 expression (Kobayashi et al., 2005). This was somewhat unexpected since TRPV1 is a heat sensor whereas TRPA1 (at least in some studies) and TRPM8 are both cold-responsive.

If TRPV1 is co-expressed with TRPA1, one has to assume that it has functional consequences. Indeed, functional interaction between these two channels was reported (Staruschenko et al., 2010), differential for neuronal subpopulations (Patil et al., 2020), and the properties of an enforced heteroconcatamer has been described (Fischer et al., 2014).

TRPV1 reporter mice allow a sensitive analysis of TRPV1 expression without dependency on antibody specificity. Reporting expression via LacZ, PLAP, or Cre-Lox systems all showed a robust TRPV1 presence in peripheral sensory neurons with good correlation to functional responses, but the percentage responding was not quantified (Wang et al., 2017). The extent of brain and extraneuronal TRPV1 expression is a controversial issue beyond the scope of this review (capsaicin-sensitive afferents), therefore the reader is referred to the literature (Mezey et al., 2000; Kauer and Gibson, 2009; Cavanaugh et al., 2011; Fernandes et al., 2012; Bevan et al., 2014; Martins et al., 2014).

TRPV1 is a useful marker of nociceptive neurons, but it is unclear what is common in these neurons beside their TRPV1 expression. Yes, TRPV1 activation can cause pain without any doubt, but there are neurons outside the TRPV1-positive populations which can transmit pain, and, conversely, there is no proof that every TRPV1-expressing neuron is nociceptive. Indeed, afferents expressing TRPV1 seem to serve distinct functions in different organs. For example, TRPV1-positive afferents in the pancreas has been implicated in the pathomechanism of diabetes; in the gastrointestinal system they were linked to thermoregulation; in the respiratory system their activation causes cough; and in the urinary bladder they alre involved in the micturition reflex (Moran et al., 2011). Another reflex pathway, the Bezold-Jarisch reflex (also known as the pulmonary chemoreflex) is also initiated by capsaicin-sensitive afferents (Harron and Kobinger, 1984). Even in the skin, TRPV1 contributes to multiple distinct sensations, as e.g., warmth and heat. The dual, sensory-afferent nature of these fibers further complicates the picture.

There is a partial overlap between TRPV1 expression and other commonly used markers in sensory pharmacology. Most, but not all, TRPV1-positive neurons are peptidergic, expressing calcitonin gene-related peptide (CGRP) and substance P (SP) (Holzer, 1988; Szallasi and Blumberg, 1999). In the mouse, peptidergic and isolectin B4-binding populations cover most sensory neurons and are mutually exclusive. However, despite clear differences in their spinal projection (Silverman and Kruger, 1990), ascribing a function to these two neurochemically distinct populations is less trivial. Therefore, these are often combined with TRPV1, which intersects differently with these populations in the mouse and rat (Dirajlal et al., 2003; Price and Flores, 2007).

With the advent of single-cell RNA sequencing, the sensory neuron populations can be clustered with high precision. Two studies analyzed mouse DRG neuron populations (Usoskin et al., 2015; Li et al., 2016) and a further study used murine trigeminal ganglia (Nguyen et al., 2017), but a synthesis of these is not trivial. Based on RNA profile, these studies described 10–17 subpopulations. In one study, TRPV1 was expressed in 6 of the 11 clusters and comprised 44% of all DRG neurons (Usoskin et al., 2015). In the second study, TRPV1 was detected in 8 of the 17 clusters, comprising 30% of all DRG neurons (Li et al., 2016). And in the third study, TRPV1 was found in 7 of the 13 clusters, comprising 50% of all trigeminal neurons (Nguyen et al., 2017). Human sensory neuron TRPV1 mRNA data have been compared to mouse (Ray et al., 2018). These data are from adult animals. In embryonic stage and in newborns, capsaicin-sensitive neurons are more widely distributed (Hjerling-Leffler et al., 2007).

Using RNA sequencing data, one may develop hypotheses about the TRPV1 responsiveness of populations which completely, partially or do not overlap with TRPV1 expression. For example, for MrgprA3 or TRPM8 there is little overlap with TRPV1, therefore at best minor changes are to be expected in response to TRPV1 activation after MrgprA3 lineage ablation. This is in line with experimental observations of an unchanged response to capsaicin (Han et al., 2013; Pogorzala et al., 2013). In contrast, NaV1.8 is largely overlapping, allowing to expect an absence of capsaicin response after NaV1.8 lineage ablation, which fits the experimental observation (Abrahamsen et al., 2008). Potential heterogeneity of the TRPV1-sensitive population can be further addressed by an intersectional approach, which eliminates a subfraction of these neurons. Ablation of CGRPα-expressing neurons reduced the time-spent licking after capsaicin injection to half, indicating that both the remaining as well as the ablated population contribute to pain-related behavior (McCoy and Zylka, 2014). Deletion of MrgprD eliminates primarily the non-peptidergic subfraction of neurons, comprising a small subset of the TRPV1-expressing neurons. Mice with MrgprD lineage ablation have been generated, but responsiveness to capsaicin has not been reported. This or a similar approach will allow in the future addressing, whether TRPV1-expressing neurons contain subpopulations, which equip the organism with distinct sensitivities.

Finally, RNA sequencing results allow an investigation of coexpressed proteins, whether they control TRPV1 expression, membrane presence and trafficking, and thereby facilitate or repress TRPV1 function.

### Activation of TRPV1 in Sensory Neurons

Activation by capsaicin of TRPV1 causes a burning pain sensation in human skin and mucosa, the intensity and duration of which can be controlled over a wide range. The discovery that TRPV1 can also be activated by noxious heat provided a mechanistic explanation for the “burning” nature of capsaicin-evoked pain. Psychophysical experimentation with capsaicin in the human oral cavity was extensively used to study desensitization and change of temperature perception (Smutzer and Devassy, 2016). In fact, Szolcsányi and coworkers used the human tongue to establish structure-activity relations for capsaicin analogs (Szolcsányi and Jancsó-Gábor, 1975). The capsaicin threshold on the tongue is 0.15–1.0 μM (Rozin et al., 1981; Sizer and Harris, 1985).

Although the use of the human tongue as experimental model went out of favor, injection of capsaicin into the skin, first described in 1987, is still a broadly used human pain model (Simone et al., 1987). The concentration-dependence of intensity and duration of capsaicin-evoked pain is well-established (Simone et al., 1989). A frequent rating, a small volume of 50 μl and a low concentration of 3.2 μM capsaicin, which is close to the threshold of the originally described model, allows a reliable pain model that lasts about one-minute (Schwarz et al., 2017). A topical capsaicin cream can also be used as a pain model, for example to test analgesic actions in humans (Lee et al., 2019).

Intraplantar capsaicin injection is an established animal pain model, with observing pain-related aversive behavior as a readout. This model was extensively used to establish target occupation and on-target analgesia by TRPV1 antagonists (Li et al., 1999; Laird et al., 2001; Massaad et al., 2004; Gavva et al., 2005). Alternatively, TRPV1 activation can also be scrutinized using the eye-wiping test (Lee et al., 2001; Bates et al., 2010).

Capsaicin-induced pain is prevented by the TRPV1 antagonists, capsazepine and BCTC. The advent of capsazepine facilitated TRPV1 research, but given inhibition of voltage-gated calcium channels (Docherty et al., 1997) and activation of TRPA1 (Kistner et al., 2016), it cannot be considered state of the art. BCTC is a potent inhibitor of TRPV1 (Valenzano et al., 2003), but turned out also to inhibit TRPM8 (Behrendt et al., 2004). This is a more acceptable flaw for use in expression systems and also for sensory fibers, as there is little overlap in expression of TRPV1 and TRPM8, as discussed above. The use of these early compounds is supplanted by potent and highly selective TRPV1 antagonists (Aghazadeh Tabrizi et al., 2017; Moran and Szallasi, 2018).

The human capsaicin-induced pain model was used to demonstrate a local axon reflex flare, primary hyperalgesia and secondary hyperalgesia (Magerl et al., 1998; Serra et al., 1998). The cellular time course of tachyphylaxis is well reflected in responses to repetitive stimulation (Witting et al., 2000). The model appears suitable to test analgesics (Wang et al., 2008).

The response of human fibers to injection of capsaicin recorded by microneurography indicated the existence of two functional populations, a mechanosensitive and a mechano-insensitive (Schmelz et al., 2000; Serra et al., 2004), with the mechanosensitive population further differentiated into subpopulations in primates (Wooten et al., 2014). However, it is unclear how these functional subpopulations map into the neuronal subpopulations defined by RNA sequencing.

Intravenous capsaicin in humans exceeding 0.5 μg/kg caused a burning sensation in chest, face, rectum and extremities (Winning et al., 1986). The majority of orally consumed capsaicin is resorbed; the pharmacokinetic and further metabolism has been investigated (Chaiyasit et al., 2009). In oral consumption, the interaction with other gustatory and olfactory stimuli is interesting. The four primary taste qualities attenuated the effects of capsaicin, but capsaicin reduced only sweet, sour and bitter but not salty taste (Lawless and Stevens, 1984; Stevens and Lawless, 1986).

### Ablation of the TRPV1-Sensitive Population

**FIGURE 1:**
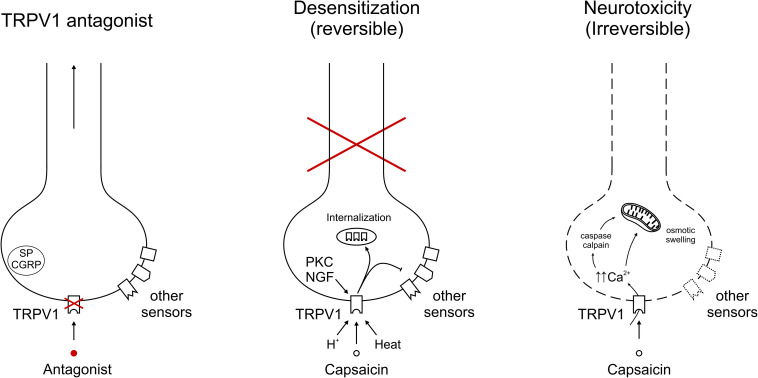
Representative depiction of TRPV1 modulation on afferent nerve endings. **(Left)** Specific TRPV1 antagonism blocks TRPV1-mediated signals, but allows transduction of the neuron through other sensors. Neuropeptides Substance P (SP) and calcitonin gene-related peptide (CGRP) indicate the potential to contribute to neurogenic inflammation. **(Middle)** Sustained and/or repeated TRPV1 gating leads to internalization of the receptor into intracellular stores, from which it is recycled or broken down. With capsaicin, heat or protons, exemplary TRPV1 agonists are depicted, in addition acute sensitization by PKC and longer-term expression regulation by NGF. The inhibitory arrow to other sensors indicates cross-desensitization of other sensors leading to a reversible inactivation of this neuron. **(Right)** TRPV1 activation leading to unsustainable intracellular calcium concentrations gives rise to irreversible damage. Mechanisms include cellular stress, e.g., through calcium, caspase and calpain activation.

TRPV1 antagonists only block TRPV1, leaving the respective neurons functional and allowing these to be activated by a number of additional pain targets. By contrast, TRPV1 agonists like capsaicin desensitize the whole neuron, rendering it “silent” (Figure 1). This explains why the capsaicin desensitization experiments overestimated the analgesic potential of TRPV1 antagonists. Furthermore, as mentioned above, capsaicin “desensitization” can be reversible and permanent, with an ill-defined line between the two.

There are multiple ways to achieve “capsaicin desensitization.” The traditional approach is injection of a TRPV1 agonist, in most studies capsaicin, with incrementally increasing doses (Jancsó and Jancsó-Gábor, 1959). The dose escalation can finally reach concentrations manyfold of the LD_50_ of naive animals. This is necessary because the therapeutic window of capsaicin is narrow, and using single doses allows only partial desensitization before respiratory depression by capsaicin kills the experimental animal (Palecek et al., 1989). Despite pulmonary dose-limiting side effects, systemic capsaicin administration leads to a critical drop of total peripheral resistance (Donnerer and Lembeck, 1982), which might be at least partially caused by the potent vasodilator CGRP released into the systemic circulation (Tang et al., 1997). The full capsaicin dose that is required for complete desensitization is usually given over a period of 5 days. In rats, desensitization can last up to 2–3 months (Jancsó et al., 1967). This is in keeping with the human experience using the topically administered high concentration capsaicin patch, Qutenza. Of note, resiniferatoxin has a much broader therapeutic window, and full desensitization can be achieved by means of a single s.c. injection (Szallasi and Blumberg, 1999).

In embryonic and in early neonatal stage, TRPV1 expression extends to a larger fraction of neurons (Hjerling-Leffler et al., 2007), therefore the fraction of capsaicin-ablated neurons is larger compared to adults (Ritter and Dinh, 1992), and there are even differences between prenatal and neonatal treatment (Perfumi and Sparapassi, 1999). The irreversible pharmacological ablation of a sensory neuronal subpopulation is still a unique and “mysterious” feature of capsaicin and its analogs.

The mechanisms underlying neurotoxicity by capsaicin remain unclear, since the so far identified mechanisms that are dependent on Ca^2+^ influx and activation of calpain and caspase are not particularly TRPV1-specific. Moreover, activation of other ion channels with high calcium conductance does not yield similar results. Adding to the confusion, neonatal capsaicin administration leads to widespread neuronal loss in the rat brain, interpreted by the investigators as hypoxic damage caused by the capsaicin-evoked respiratory arrest (Ritter and Dinh, 1992).

Ablation of the TRPV1-lineage is also possible by expressing the diphtheria toxin receptor as a cell death switch under the TRPV1 promoter. Injecting the diphtheria toxin in such animals allows ablation of the TRPV1-expressing neurons (Baral et al., 2018).

A further approach to investigate the TRPV1 lineage is a functional silencing, which has been described for the charged and membrane impermeable sodium channel blocker, QX314. Coadministration with a TRPV1 agonist allows selective uptake of QX314 through the ion channel pore of the TRPV1 channel (Brenneis et al., 2013). The treated animals showed deficits in heat and mechanical pressure, but not in pinprick and touch detection. They also showed reduced inflammatory hyperalgesia. However, QX314 also permeates the ion channel pore of TRPA1 (Stueber et al., 2016) which this limits specificity.

So, what are the main differences between *Trpv1* knockout animals and those with “silenced” TRPV1 lineage? *Trpv1* knockouts show only minor defects in physiological heat sensation (Caterina et al., 2000). In contrast, ablation of the whole TRPV1 lineage by diphtheria toxin eliminated withdrawal from a 55°C hot plate within the test cutoff values (Mishra et al., 2011). Profound thermal hypoalgesia was also noted in rats following resiniferatoxin administration (Xu et al., 1997; Bölcskei et al., 2010). There was also a strong effect on the thermal preference, with both extreme hot and cold temperatures largely ignored by these animals (Pogorzala et al., 2013). In humans, desensitization by capsaicin elevated the heat detection threshold and reduced suprathreshold pain (Rosenberger et al., 2020). The difference between eliminating the TRPV1 lineage and only TRPV1 suggested the presence of further heat sensing ion channels in neurons expressing TRPV1. Based on the observations, one may argue that TRPV1 plays only a minor role in physiological heat sensation of rodents, and the additional channels involved in mice were unexpected (Vandewauw et al., 2018). In contrast, TRPV1 inhibition in human subjects clearly elevated the heat thresholds, therefore TRPV1 acts a first line of defense against acute non-damaging heat (Arendt-Nielsen et al., 2016; Manitpisitkul et al., 2018). Interestingly, modality specific TRPV1 antagonist NEO6860, which does not block heat activation *in vitro*, did also not alter human heat thresholds (Brown et al., 2017). The behavioral response to “inflammatory soup” (that contains ATP, prostaglandins, bradykinin, histamine, and serotonin) was markedly reduced after TRPV1 lineage-ablation; however, this misses a direct comparison to the *Trpv1* knockout as a relevant fraction of inflammatory sensitisation converges on TRPV1 (Caterina et al., 2000). Pain-related behavior induced by ATP was also reduced after TRPV1 lineage-ablation, indicating that the involved ATP receptors substantially overlap with TRPV1. The resting body temperature was not different after TRPV1-lineage ablation, but the counterregulation to thermal stress as well as the induction of fever by interleukin-1β was reduced. The TRPV1 lineage was also required for ongoing inflammatory pain induced by carrageenan, but TRPV1 inhibition could not antagonize this as resiniferatoxin pretreatment (Okun et al., 2011). Comparison of RNA sequencing of TRPV1-expressing neurons and the complementary population shows the relative abundance of many established pain targets (Goswami et al., 2014).

Topical capsaicin desensitization has a clear therapeutic potential to relieve pain, but has also mysterious inconsistencies. In fact, over-the-counter capsaicin creams are broadly available for muscle and arthritic pain, though their analgesic value is only marginally better than placebo (Szallasi and Blumberg, 1999). To increase clinical efficacy, site-specific capsaicin injections and dermal patches have been developed. Qutenza is a dermal patch (capsaicin, 8% w/w), indicated for neuropathic pain (Blair, 2018). It provides a variable, but clinically meaningful improvement in neuropathic pain patients for about 3 months (Martini et al., 2012; Wagner et al., 2013; Maihöfner and Heskamp, 2014).

For decades, sensitivity to capsaicin was recognized as a functional signature of primary sensory neurons. Indeed, for concentrations of up to 1 μM there is little evidence for non-specific, TRPV1-independent capsaicin actions. At 10 μM and above, however, capsaicin loses its selectivity for TRPV1 and starts interacting with various enzymes, changing membrane fluidity, and blocking other receptors (Holzer, 1991; Szallasi and Blumberg, 1999; Braga Ferreira et al., 2020). Indeed, cell death evoked by capsaicin 100 μM is well documented in *Trpv1* knockout mice (Yang et al., 2014). When the clinical experience with high concentration capsaicin preparations is puzzling, the possibility that excessive capsaicin concentrations may also have acted on targets other than TRPV1 should be considered.

First, there is the high capsaicin concentration in the Qutenza patch. Acute desensitization can be achieved by low concentration (0.02–0.15% are common) capsaicin cremes (Derry and Moore, 2012), but a 1% dermal patch is less efficacious than the 8% patch to achieve a reversible “cutaneous nerve terminal axotomy”. The variable delivery through the skin and the systemic redistribution might only partially explain why Qutenza contains a concentration which is orders of magnitude (by about 4 × 10^6^) higher than the EC_50_ of capsaicin at human TRPV1. Data on transdermal delivery indicate that within one hour about 1% of the capsaicin contained in the Qutenza patch is delivered (Wohlrab et al., 2015). A potential explanation for the required high concentration might be a need to reach intracellular TRPV1 (Zhao and Tsang, 2017), about 100-fold higher capsaicin concentrations compared to the plasma membrane were required for a half-maximal effect (Gallego-Sandín et al., 2009).

Second, in Qutenza-treated patients, the density of TRPV1-positive dermal fibers was reduced with corresponding reduction in the detection of touch and pinprick stimuli, but not heat sensation, which is at odds with expectations based on animal data obtained with TRPV1 ablation (Kennedy et al., 2010).

Third, the duration of clinical pain relief clearly exceeds the recovery of skin sensitivity, which is largely normalized after 21 days (Lo Vecchio et al., 2018).

Fourth, the Qutenza effect differs between patients and healthy volunteers. The induced pain in healthy volunteers shows high variability, including subjects classified as non-responders, of which many had barely any capsaicin patch-induced pain (Gustorff et al., 2013; Papagianni et al., 2018). The capsaicin patch may affect Aδ-fiber function in humans, and the extent was correlated with pain reduction by the capsaicin patch (Papagianni et al., 2018). Expression of TRPV1 in Aδ-fibers during disease states, but not under physiological conditions might serve as explanation.

Finally, one has to keep in mind that for Qutenza to be clinically effective, the treated patient must have functional capsaicin-sensitive nerves, which may not be the case for some individuals. For example, patients with advanced diabetes may lose most of their TRPV1+ afferents as implied by a murine model of diabetic peripheral neuropathy (Pabbidi et al., 2008).

## Concluding Thoughts: The Mysteries of Capsaicin-Sensitive Afferents

Capsaicin research has a long and rich history. In dogs, Hõgyes reported the hypothermic action of intragastric pepper extract in 1878 as the first indication of the role that TRPV1-positive afferents play in thermoregulation (Högyes, 1878). Yet, almost 150 years later, the exact mechanism by which TRPV1 regulates body temperature remains a mystery. Early studies with microinjection of capsaicin into brain nuclei pointed to the existence of a capsaicin-responsive thermoregulation center in the preoptic area (Jancsó-Gábor et al., 1970). By contrast, results obtained with peripherally restricted TRPV1 antagonists implicated a peripheral target (Tamayo et al., 2008). Other studies (Szolcsányi, 2015) argued that capsaicin “tricks” animals into feeling hot, and these animals try to cool down by seeking out cold surfaces. Indeed, capsaicin-treated rodents have red ears due to vasodilation, and they spread out in their cages. Moreover, dogs pant and humans sweat in response to capsaicin. However, rats desensitized to capsaicin suffer heat stroke when moved to a heat chamber, implying that capsaicin may impair thermoregulation instead of simply causing hypothermia (Jancsó-Gábor et al., 1970).

TRPV1 agonists and antagonists induce opposite changes in body temperature, hypo- and hyperthermia, respectively. Based on these observations, it was proposed that TRPV1 has an endogenous tone (Gavva, 2008). This model located TRPV1+ afferents responsible for body temperature changes in the gastrointestinal tract. Yet, ablation by resiniferatoxin of the abdominal TRPV1-expressing afferents did not eliminate the capsaicin-sensitive thermoregulatory response (Szolcsányi, 2015). Adding to the confusion, global *Trpv1* knockout mice have normal body temperature (Szelényi et al., 2004). Though one may argue that these animals may have developed alternative thermoregulatory pathways to compensate for the missing TRPV1.

Studies with TRPV1 antagonists further clouded the picture. It turned out that some antagonists caused a febrile reaction in human volunteers whereas others did not change body temperature perceptibly or, paradoxically, they even lowered body temperature [reviewed in Garami et al. (2020)]. Although rational drug design resulted in “temperature neutral” TRPV1 antagonists (substances that did not block proton activation did not cause hyperthermia either), some of these compounds proved “rat-specific” as they still caused hyperthermia in dogs and primates (Lehto et al., 2008). Nevertheless, with NEO6860 there is a TRPV1 antagonist which does not invoke hyperthermia in humans (Brown et al., 2017). As an interesting twist, the undesirable hyperthermia by TRPV1 antagonists in pain patients might even be useful in patients with surgically-induced hypothermia (Schmidt, 2017).

Although TRPV1 is clearly a heat-activated channel with an activation threshold about 40°C (Caterina et al., 1997), the role of TRPV1 in physiological heat sensation remains a mystery. It was suggested that TRPV1 is really a warmth receptor (Yarmolinsky et al., 2016), though the burn injuries in patients taking TRPV1 antagonists argue otherwise. Species-related differences should also be taken into consideration. For example, camel TRPV1 is not heat-sensitive (Laursen et al., 2016). Rats have heat-sensitive TRPV1 channels, but mole rats living in burrows underground do not.

But maybe the biggest, and probably most important, mystery of all is the discrepancy between the therapeutic potential of TRPV1 antagonists in preclinical models and clinical studies. In experimental animals, TRPV1 antagonists blocked inflammatory, neuropathic, and cancer pain (Szallasi et al., 2007; Moran et al., 2011; Moran and Szallasi, 2018). However, in patients with migraine and osteoarthritis, TRPV1 inhibition disappointed as therapeutic approach (Mayorga et al., 2017; Arsenault et al., 2018; Vécsei et al., 2019). Likewise, TRPV1 antagonists inhibited evoked cough in experimental animals, but did not show any antitussive activity in chronic cough patients (Khalid et al., 2014; Belvisi et al., 2017).

The explanation of the discrepant preclinical and clinical TRPV1 antagonist efficacies remains to be elucidated. Maybe TRPV1 plays a much less important role in human diseases than in their animal models. Or maybe one size simply does not fit all: one ought to select the patient population that could benefit from the TRPV1 antagonist therapy (targeted approach).

In conclusion, capsaicin has been an invaluable tool in sensory pharmacology to dissect a fundamental population of sensory afferents, and to explore their functions in health and disease. The capsaicin receptor was identified as TRPV1, and a number of selective and potent small molecule antagonists developed. Despite the tremendous progress in our understanding of TRPV1 mechanisms, several “mysteries” remain, ranging from the molecular mechanisms of capsaicin desensitization through the exact role of TRPV1 in thermoregulation and heat sensation to the therapeutic value of TRPV1 antagonists.

## Author Contributions

All authors listed have made a substantial, direct and intellectual contribution to the work, and approved it for publication.

## Conflict of Interest

The authors declare that the research was conducted in the absence of any commercial or financial relationships that could be construed as a potential conflict of interest.
